# Process evaluation of electron beam irradiation-based biodegradation relevant to lignocellulose bioconversion

**DOI:** 10.1186/2193-1801-3-487

**Published:** 2014-08-29

**Authors:** Jin Seop Bak

**Affiliations:** Department of Chemical and Biomolecular Engineering, Advanced Biomass R&D Center, KAIST, 291 Daehak-ro, Yuseong-gu, Daejeon, 305-701 Republic of Korea

**Keywords:** Bioethanol, Biomass pretreatment, Electron beam irradiation-based biodegradation, Lignocellulose, Transcriptomics

## Abstract

In order to solve the inefficient problem of long-term biodegradation by wood-decaying fungus, rice straw (RS) was depolymerized using electron beam irradiation-based biodegradation (EBIBB). This environment-friendly program without the use of inhibitory byproducts significantly increased the digestibility and fermentability of RS. Specifically, when irradiated RS was simultaneously biodegraded by *Phanerochaete chrysosporium* for 10 days, the sugar yield was 65.5% of the theoretical maximum. This value was on the same level as the 64.8% (for 15 days) measured from unirradiated RS. In case of fermentability, similarly, EBIBB program had an effect on time/energy saving. Furthermore, the transcriptomic profiles under different biosystem were analyzed in order to verify possible substrate-specific regulation based on change of lignocellulosic components. Interestingly, the overall correlation based on the bias (upregulation or downregulation) was reasonably analogous, especially lignocellulolysis-related genes.

**Electronic supplementary material:**

The online version of this article (doi:10.1186/2193-1801-3-487) contains supplementary material, which is available to authorized users.

## Introduction

Fuel bioethanol from lignocellulosic plant biomass is being explored as an alternative energy source due to exhaustion of oil resources and environmental concerns, especially global warming. To commercialize bioethanol process, the effective pretreatment of the biomass are essential, due to the inaccessibility from cellulose crystallinity, in the bioconversion of recalcitrant substrates into fermentable sugars (Sanderson, 2011). Recent trends of pretreatment process have been studied on environmentally friendly biodegradations using wood-rotting fungi instead of general processes using physicochemical tools and evaluated by various indexes (Menon and Rao, 2012; Wan and Li, 2012). However, the use of only biodegradation to enhance the hydrolysis yield of lignocellulosic substrates has not been sufficient for commercial programs yet. More importantly, it is hard for useful programs to hydrolyze the substrates due to the inevitable necessity of long-term treatment.

Electron beam technology have been broadly studied to extend the range of applications in the properties of the polymeric materials (Hamm and Hamm, 2012). Particularly, the mechanism of chain scission (by electron attacks) focus on change (or degradation) in structural crystallinity of substrates (e.g., lignocellulose; Bak, 2014b). Therefore, to address the weak points in the fungal biodegradation, such as the low yield and the long-term process, an irradiation-treated substrate was used in this biodegradation program. This study was conducted to verify the feasibility and efficiency of electron beam irradiation-based biodegradation (EBIBB) program. Its impact was evaluated based on various bioprocessing properties of pretreated substrate, such as digestibility yield and fermentation efficiency. Furthermore, in order to understand the mainstream of fungal lignocellulolytic system in EBIBB program, the pattern of gene expression profiles was analyzed using whole genome microarray-based approach at transcriptome level.

## Materials and methods

### Electron beam irradiation-based biodegradation system

Rice straw (RS), harvested from Korea University Farm (Deokso, Korea), was used as the lignocellulose model compound. After the preprocessing procedures (Supporting Information), processed RS was used as the starter substrate for the fungal biodegradation. Prior to the biodegradation, RS was irradiated by using a linear electron accelerator (Korea Atomic Energy Research Institute, Daejeon, Korea) in order to enhance the effects of substrate pretreatment. The optimized condition (1 Mev and 80 kGy at 0.12 mA) of irradiation was based on a previously reported methodology (Bak et al., 2009b).

Next, based on previously optimized fungal cultivation (Bak et al., 2009a), after the addition of irradiated RS (4.4 g), *Phanerochaete chrysosporium* (ATCC 32629) was cultured in 200 mL of optimized medium containing 1% (w/v) of glucose (as an initial carbon source) at 29°C and 150 rpm for 15 days. No substrate was added to the control cultures. Further details are provided in Additional file 1.

### Downstream evaluation

Simultaneously, the concentration of inhibitory byproducts (hydroxymethylfurfural and furfural) and theoretical maximum yields (digestibility and fermentability) of the EBIBB-pretreated RS were analyzed following the public biomass analytical protocols (http://www.nrel.gov/biomass/capabilities.html). Further details are provided in Additional file 1. At the same time, according to the public biomass protocols, the change of 3 components (lignin, cellulose, and hemicellulose) of RS were confirmed based on a dry weight basis. Based on generally accepted methods (Additional file 1), the extracellular activities of well-known enzymes involved in lignocellulose degradation were assayed during the biodegradation. Further details are provided in Additional file 1. All experiments were conducted in triplicate.

After EBIBB pretreatment, the microstructural changes of substrates was determined using a Hitachi S-4700 scanning electron microscope (Tokyo, Japan). Especially, diffraction spectra of substrates was performed with a powder X-ray diffractometer (Bruker D5005, Karlsruhe, Germany) to identify crystallinity index on EBIBB-pretreated RS. The signals were analyzed in triplicate using the previously confirmed θ-2θ method (Bak et al., 2009b).

### Upstream evaluation

Under different biodegradation condition (whether irradiation-based system or not), the complementary relationship between the lignocellulolytic targets and % theoretical yields was analyzed by transcriptomic expression analysis. After six biological replicates of the biodegradations, cDNA hybridization of targets was performed with Custom Array 12 K microarray (CombiMatrix Corporation, Mukilteo, WA). The significance of the array was confirmed with the quantitative real-time PCR data. Further details are provided in Additional file 1. After the data processing (Additional file 1), hierarchical clustering was performed to reorganize genes into functional categories (Eisen et al., 1998). In order to graphically present the genetic expression, PermutMatrix ver. 1.9.3 software (http://www.atgc-montpellier.fr/permutmatrix/) was used in this study (Caraux and Pinloche, 2005).

## Results and discussion

### Theoretical yields of EBIBB system

**Figure 1:**
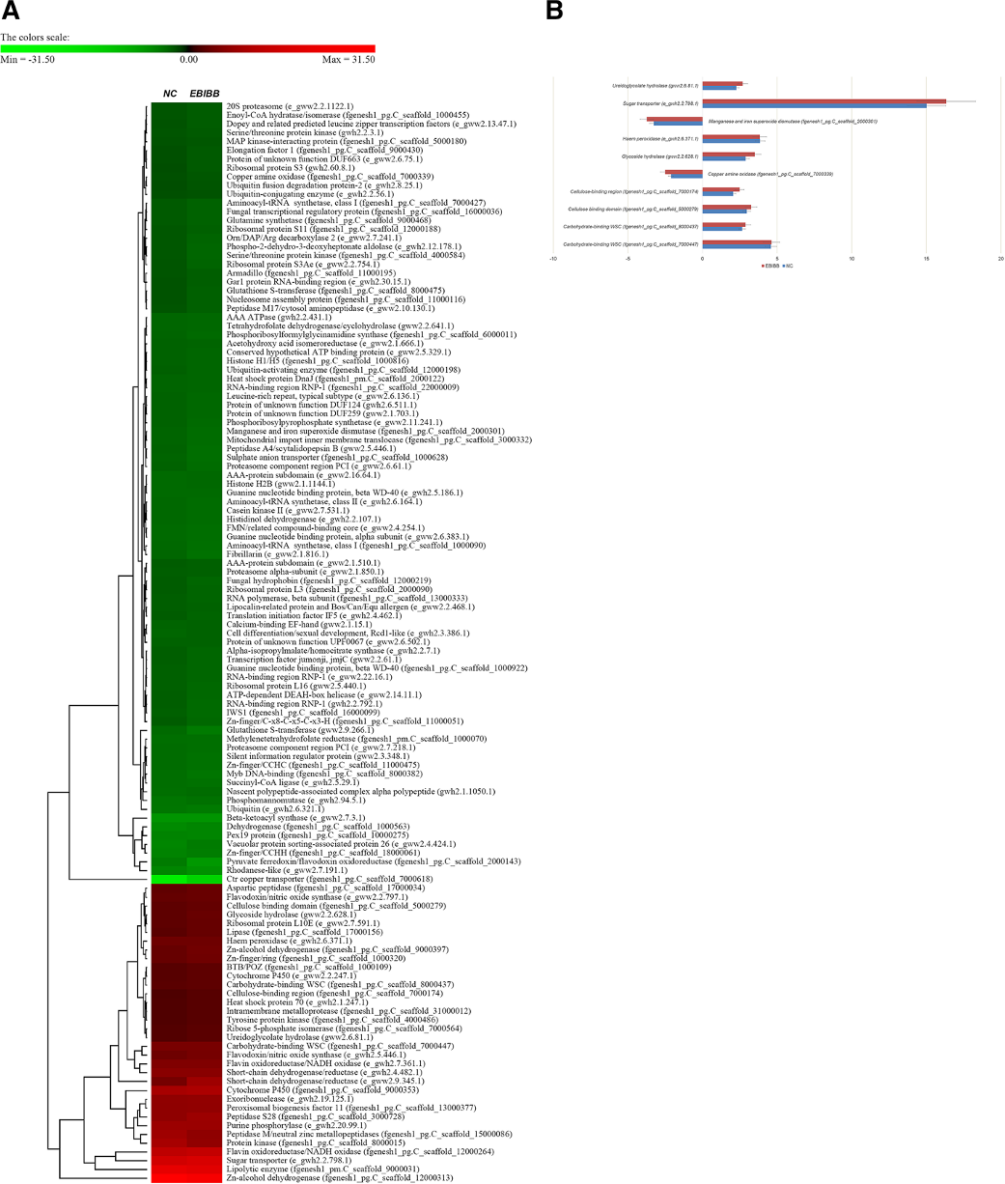
**Transcriptome profiles of** ***P. chrysosporium*** **in advanced EBIBB program. (A)** Hierarchical clustering of 123 targets showing significant differences in expression with *P* < 0.05 and |fold change| > 2 in EBIBB and NC (negative control; biodegradation without the irradiation) cultures. Lanes EBIBB and NC depict 10 days and 15 days respectively from culture grown on RS. Putative functions of the significant factors based on the U.S. Department of Energy Joint Genome Institute public database. **(B)** Change pattern of 10 targets involved in lignocellulolytic cascades.

In order to evaluate the digestibility of EBIBB treatment, the treated RS was simultaneously hydrolyzed by the addition of both β-glucosidase and cellulase. Sugar indexes (after 96 h; stationary phase) were 55.2% and 65.5% from treated RS with biodegradation periods of 5 days and 10 days, respectively (Table 1). However, increasing the degradation period from 10 days to 15 days did not predominantly increase the yield. Probably, this phenomenon may have been higher during the uptake of glucose by fermentable fungus than the release of glucose from the substrate. Furthermore, remarkably, regarding the limitation (below 70%) of maximum yield, it implies that cellular stability have significant effect on biodegradation efficiency, which predicts at active control-based compensatory metabolisms (e.g., stress-response pathways and secondary metabolisms; Figure 1A) to maintain cellular homeostasis, regardless the difference of external circumstance. As the effect of irradiation has shown, the digestibility of unirradiated RS was just 64.8% of the maximum sugar yield regardless of long-term cultivation (over 15 days) (Table 1). Probably, the modified structure of polymeric compounds by the radicals from the electrons may accelerate to the access of lignocellulolytic enzymes, and thus can shorten a lengthy time of biodegradation program for % yield maximum (Chen and Dixon, 2007). Interestingly, the fermentability (after 72 h; stationary phase) from the EBIBB system was approximately 2.1 times higher than that of untreated sample, which is likely due to the activation (Figure 1B) of cellulolytic cascades based on the open structure of pretreated lignocellulose. When only biodegradation was treated, the maximal yield was determined to be 62.5% in spite of long-term fermentation of 15 days. Regarding the generation of inhibitory compounds against the EBIBB system, although the yields of the EBIBB-RS were lower than those of biomass pretreated using conventional tools, the main inhibitors, such as acetic acid, HMF, and furfural, was either negligible or not detected. In terms of theoretical yields, the accumulation of the inhibitors under the physicochemical conventional system (especially dilute acid treatment) was found to result in lower bioconversion compared to substrate utilization on nature-friendly system (Merino and Cherry, 2007).

**Table 1:** **Downstream evaluation for scale-up in advanced EBIBB program**

Type	Inhibitory byproducts			Hydrolysis index ^d^(per 100 g biomass)			Fermentation index ^e^(per 100 g biomass)
	HMF ^c^(w/w, %)	Furfural ^c^(w/w, %)	Acetate(g/L)	EBIBB for 5 days	EBIBB for 10 days	EBIBB for 15 days	EBIBB after 10 days
EBIBB	Not detected	Not detected	< 0.05	≤ 55.2% (17.3 ± 0.2 g glucose)	≤ 65.5% (20.7 ± 0.1 g glucose)	≤ 61.2% (19.1 ± 0.2 g glucose)	≤ 62.9% (10.0 ± 0.2 g ethanol) at 10 day
NC^a^	Not detected	Not detected	< 0.02	≤ 48.3% (15.4 ± 0.2 g glucose)	≤ 58.4% (18.7 ± 0.2 g glucose)	≤ 64.8% (20.8 ± 0.2 g glucose)	≤ 62.5% (9.9 ± 0.2 g ethanol) at 15 day
Untreated^b^	Not detected	Not detected	< 0.04	≤ 27.1%^b^ (8.7 ± 0.1 g glucose)			≤ 29.5%^b^ (4.7 ± 0.1 g ethanol)

^a^negative control; biodegradation without the irradiation.

^b^without either the EBI or the biodegradation.

^c^determined as  and

^d^the yield of theoretical maximum glucose based on the enzymatic digestibility after 96 h.

^e^the yield of theoretical maximum ethanol based on the simultaneous saccharification and fermentation after 72 h.

**Figure 2:**
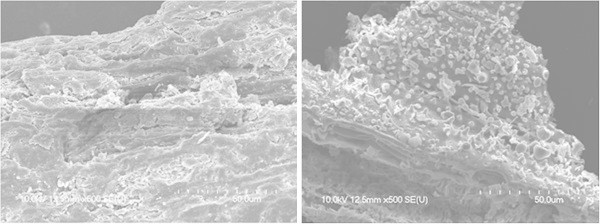
**Scanning electron microscopic image of RS substrate that was pretreated by EBIBB program.** (Left) Untreated RS (500× magnified). (Right) RS pretreated by optimal EBIBB for 10 days (500× magnified).

Overall, the hydrolysis yield of EBIBB program, which is reflected in fermentable sugars, was lower than those of plant biomass (70-85%) pretreated using conventional chemical program (Kim et al., 2002; Ko et al., 2009). Furthermore, the ethanol productivity (0.52 g/L/h) of chemical programs (especially ammonia-soaking treatment) are greater than the productivity (0.40 g/L/h) observed in the present study. However, we speculate that this program is superior to others, especially alkaline (30-80%) and fungal-based program (<50% after 14 days), in terms of the% yield and time effectiveness (Keller et al., 2003; Shi et al., 2009; Zhao et al., 2008). Additionally, the fermentability (<0.03 g ethanol/g lignocellulosic biomass) in previous *P. chrysosporium*-based system was finally obtained after 144 h of fermentation (Shi et al., 2009; Shrestha et al. 2008), which was not more than the EBIBB-level (0.10 g ethanol/g RS after 72 h). More importantly, unlike previous system, the EBIBB approach secured a bridgehead for energy/time saving in conventional competitiveness.

### Change of lignocellulosic structure

Unlike the smooth structure of untreated lignocellulosic surfaces, EBIBB-pretreated surfaces had randomly degraded cracks and non-spherical protrusions (Figure 2). We speculate that the exposure of crystalline structures may be further accelerated by inducible cell-wall disruption due to EBI-based preprocess. However, when compared to non-EBI biodegradation (Bak et al., 2009a), the change in the crystalline (or amorphous) structures were hard to distinguish by EBIBB within the significant difference.

Regarding the internal components in EBIBB-RS, the changes in total mass were negligible to within an error range (Table 2). However, the 3 major components of EBIBB-based RS showed significant reductions of mass compared to those of the original biodegradation. The formation of radicals may have accelerated a direct attack to an external layer composed of polymeric complexes, if EBI pretreatment helps to loosen the lignin (or polysaccharide) wall, then extracellular lignocellulolytic enzymes have more space for extensive participation. Loss of the recalcitrant materials can also confirm, in various conventional pretreatments, that the loss of them is different in the initial content (Sun and Cheng, 2002).

**Table 2:** **Analysis of the main components of RS following EBIBB pretreatment**

Type	Total external substrate ^c^ (dry wt. basis)	Change of RS components		
		Lignin (g lignin/L) Before/After	Cellulose (g glucan/L) Before/After	Hemicellulose (g xylan/L) Before/After
EBIBB (at 10 days)	21.5 g RS^d^ (97.7%)	4.1 g/≤ 2.6 g	7.8 g/≤ 6.1 g	2.3 g/≤ 1.4 g
NC^a^ (at 15 days)	22.0 g RS (100.0%)	4.4 g/≤ 3.4 g	7.9 g/≤ 6.5 g	2.4 g/≤ 1.9 g
Untreated^b^	22.0 g RS (100.0%)	4.4 g/4.4 g	7.9 g/7.9 g	2.4 g/2.4 g

^a^negative control; biodegradation without the irradiation.

^b^without either the EBI or the biodegradation.

^c^total amount of initially added RS before the biodegradation.

^d^loss (below 2.3%) of RS substrate by the EBI.

### Transcriptomic evaluation of irradiation-based fungal biosystem

In the biodegradation system of lignocellulosic biomass, cooperation and harmony of genetic factors is an indispensable feature for evolutionary survival tactic (Cullen and Kersten, 2004). Furthermore, it means that the effective yields of biodegradation may well have involved the systematic regulation of upstream signals (especially lignocellulolytic genes).

**Figure 3:**
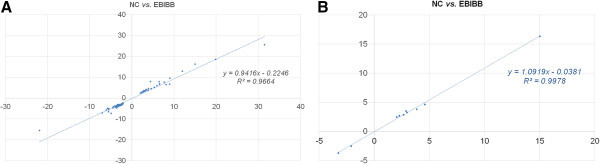
**Correlation analysis between EBIBB and NC (negative control; only biodegradation) based on the logarithmic intensities (|fold change| > 2 and** ***P*** **< 0.05) from cDNA hybridization.** RNA was directly sampled from fungal mycelial pellets grown on EBIBB for 10 days or NC for 15 days. **(A)** Significant full genes. **(B)** Lignocellulolysis-related genes.

In order to understand the internal mechanism of optimized depolymerization by *P. chrysosporium*, the expression pattern of biodegradation-regulated genes was inferred based on the comprehensive analysis of transcriptome profile (Figure 1). Regardless of either irradiation-treatment or degradation-period, interestingly, the profiles of all targets involved in intracellular regulatory and metabolic system (generally downregulated) were generally similar (Figures 1A and 3). Under drastic starvation (or the presence of recalcitrant substrates), it means that fungal cells should not make any more a needless waste of metabolic pathways, probably due to the homeostasis to fulfill the heavy energy expenditure. Furthermore, self-regulated fungal biodegradation may maintain their stability and effectiveness as a complementary manner (Bak, 2014a). In addition, the demand of cellular equilibrium and defense against the external stresses may not keep any more lignocellulolytic convergence, and thus can be predicted in the limited improvement of % theoretical yield (Figure 1 and Table 1).

Based on fungal transcriptome data, independent of the irradiation treatment, we confirmed that lignin modification may be occurred via the activation of radical-based systems (especially by peroxidases) (Figure 1B). Furthermore, carbohydrate-active enzymes (CAZys; especially binding domains, glycoside-hydrolases, and transporters) as highly essential hydrolyzable factors were responsible for the conversion of preprocessed substrates into monomeric sugar and ethanol. Remarkably, when compared to the prominent activities of aggressive targets in the context of ligninolysis, the induction of CAZys (especially glucosidase, cellobiose dehydrogenase, and xylanase; core release factors for downstream sugar compounds) in EBIBB had a very low utilization rate (|fold| < 2), unlike previously reported wood-degrading systems (Fernandez-Fueyo et al., 2012; Vanden Wymelenberg et al., 2010). This is probably due to the advanced program (via target optimization) in view of efficient biodegradation yield. It just may be partially inclusive of core factors in aggressive ligninolysis (within the limit of homeostatic system). However, extracellular fungal biosystem did not still rule out the minor factors which support the optimized yield on either sugar recovery or ligninolysis. Simultaneously, the well-known extracellular targets of lignocellulolytic mechanism extensively activated in both biosystem (Table 3). The effect of extracellular cascades were also supported by the losses of major solid components (glucan, xylan, and lignin) of RS as well as the enhancement of both hydrolysis and fermentation yield (Table 1 and 2).

**Table 3:** **Extracellular activity of well-known linocellulolytic targets in optimal EBIBB system**

Type	Ligninolytic enzymes (U/L)				Cellulolytic enzymes (U/L)			Ligninolytic oxalate (g/L)
	Lignin peroxidase	Manganese peroxidase	Glyoxal oxidase	Aryl-alcohol oxidase	β-glucosidase	Cellobiose dehydrogenase	Xylanase	
EBIBB (at 10 days)	480–650	900–1,200	≤ 350	≤ 130	≤ 140	≤ 65	≤ 435,000	≤ 0.15
NC^a^ (at 15 days)	500–700	1,100–1,500	≤ 400	≤ 180	≤ 120	≤ 55	≤ 420,000	≤ 0.20
Untreated^b^	Not detected	Not detected	Not detected	Not detected	Not detected	Not detected	Not detected	Not detected

^a^negative control; biodegradation without the irradiation.

^b^without either the EBI or the biodegradation.

Based on the results of above mentioned similarity (here transcriptomic expression), we can predict that the abundance (or presence) of opened (or modified) biodegradable substrates (by directly oxidative attack of electrons) is a key to the understanding of *P. chrysosporium* metabolism. In other words, an important determinant of mainstream (or substream) in fungal biodegradation mechanism is really a matter of substrate style (structure and component; Figure 2 and Table 2) rather than just recalcitrant substrate. Furthermore, we confirmed that the combined program containing the irradiation treatment help to enhance the functional metabolic uniformity in the bioconversion process (or regulatory network).

## Conclusions

Based on mass balance, the EBIBB-pretreated RS after 10 days showed significant increases in industrial yields compared to the untreated RS. Particularly, the reduction of a lengthy time in advanced EBIBB-program had a strong advantage in downstream bioprocess. Although the production yields of this program was lower than those of substrate pretreated by physicochemical programs, the inhibitory byproducts was rarely generated. Microfibril composition analysis revealed that physical (or chemical) changes in substrate surfaces were likely a result of EBIBB. Lastly, the profiling of intracellular genes involved in lignocellulolytic cascades during the optimal EBIBB-treatment could help the understanding of mainstream system.

## Electronic supplementary material

Additional file 1: **Supporting Information.** (DOC 68 KB)

## References

[CR1] Bak JS (2014). Complementary substrate-selectivity of metabolic adaptive convergence in the lignocellulolytic performance by Dichomitus squalens. Microbiol Biotechnol.

[CR2] Bak JS (2014). Electron beam irradiation enhances the digestibility and fermentation yield of water-soaked lignocellulosic biomass. Biotechnol Rep.

[CR3] Bak JS, Ko JK, Choi IG, Park YC, Seo JH, Kim KH (2009). Fungal pretreatment of lignocellulose by *Phanerochaete chrysosporium* to produce ethanol from rice straw. Biotechnol Bioeng.

[CR4] Bak JS, Ko JK, Han YH, Lee BC, Choi IG, Kim KH (2009). Improved enzymatic hydrolysis yield of rice straw using electron beam irradiation pretreatment. Bioresour Technol.

[CR5] Caraux G, Pinloche S (2005). Permutmatrix: a graphical environment to arrange gene expression profiles in optimal linear order. Bioinformatics.

[CR6] Chen F, Dixon RA (2007). Lignin modification improves fermentable sugar yields for biofuel production. Nat Biotechnol.

[CR7] Cullen D, Kersten PJ, Brambl R, Marzluf GA (2004). Enzymology and molecular biology of lignin degradtion. The mycota III: biochemistry and molecular biology.

[CR8] Eisen MB, Spellman PT, Brown PO, Botstein D (1998). Cluster analysis and display of genome-wide expression patterns. Proc Natl Acad Sci U S A.

[CR9] Fernandez-Fueyo E, Ruiz-Dueñas FJ, Ferreira P, Floudas D, Hibbett DS, Canessa P, Larrondo LF, James TY, Seelenfreund D, Lobos S, Polanco R, Tello M, Honda Y, Watanabe T, Watanabe T, Ryu JS, Kubicek CP, Schmoll M, Gaskell J, Hammel KE, St John FJ, Vanden Wymelenberg A, Sabat G, Splinter BonDurant S, Syed K, Yadav JS, Doddapaneni H, Subramanian V, Lavín JL, Oguiza JA (2012). Comparative genomics of *Ceriporiopsis subvermispora* and *Phanerochaete chrysosporium* provide insight into selective ligninolysis. Proc Natl Acad Sci U S A.

[CR10] Hamm RW, Hamm ME (2012). Industrial accelerators and their applications.

[CR11] Keller FA, Hamilton JE, Nguyen QA (2003). Microbial pretreatment of biomass: potential for reducing severity of thermochemical biomass pretreatment. Appl Biochem Biotechnol.

[CR12] Kim KH, Tucker MP, Nguyen QA (2002). Effects of pressing lignocellulosic biomass on sugar yield in two-stage dilute-acid hydrolysis process. Biotechnol Prog.

[CR13] Ko JK, Bak JS, Jung MW, Lee HJ, Choi IG, Kim TH, Kim KH (2009). Ethanol production from rice straw using optimized aqueous-ammonia soaking pretreatment and simultaneous saccharification and fermentation processes. Bioresour Technol.

[CR14] Menon V, Rao M (2012). Trends in bioconversion of lignocellulose: biofuels, platform chemicals & biorefinery concept. Prog Energy Combust Sci.

[CR15] Merino ST, Cherry J (2007). Progress and challenges in enzyme development for biomass utilization. Adv Biochem Eng Biotechnol.

[CR16] Sanderson K (2011). Lignocellulose: a chewy problem. Nature.

[CR17] Shi J, Sharma-Shivappa RR, Chinn M, Howell N (2009). Effect of microbial pretreatment on enzymatic hydrolysis and fermentation of cotton stalks for ethanol production. Biomass Bioenergy.

[CR18] Shrestha P, Rasmussen M, Khanal SK, Pometto AL, Van Leeuwen JH (2008). Solid-substrate fermentation of corn fiber by *Phanerochaete chrysosporium* and subsequent fermentation of hydrolysate into ethanol. J Agric Food Chem.

[CR19] Sun Y, Cheng J (2002). Hydrolysis of lignocellulosic materials for ethanol production: a review. Bioresour Technol.

[CR20] Vanden Wymelenberg A, Gaskell J, Mozuch M, Sabat G, Ralph J, Skyba O, Mansfield SD, Blanchette RA, Martinez D, Grigoriev I, Kersten PJ, Cullen D (2010). Comparative transcriptome and secretome analysis of wood decay fungi *Postia placenta* and *Phanerochaete chrysosporium*. Appl Environ Microbiol.

[CR21] Wan C, Li Y (2012). Fungal pretreatment of lignocellulosic biomass. Biotechnol Advances.

[CR22] Zhao Y, Wang Y, Zhu JY, Ragauskas A, Deng Y (2008). Enhanced enzymatic hydrolysis of spruce by alkaline pretreatment at low temperature. Biotechnol Bioeng.

