# Supplementary material for: Process evaluation of electron beam irradiation-based biodegradation relevant to lignocellulose bioconversion
Source: Springerplus. 2014 Aug 29;3:487. doi: 10.1186/2193-1801-3-487 (PMC4164673; doi:10.1186/2193-1801-3-487)
Supplement: Supplementary file 1 — Additional file 1: Supporting Information. (DOC 68 KB) [file 40064_2014_1201_MOESM1_ESM.doc]

**Supporting Information**

Process evaluation of electron beam irradiation-based biodegradation relevant to lignocellulose bioconversion

Jin Seop Bak

*Department of Chemical and Biomolecular Engineering, Advanced Biomass R&D Center, KAIST, 291 Daehak-ro, Yuseong-gu, Daejeon, 305-701, South Korea*

*Address correspondence to Jin Seop Bak, jsbwvav7@kaist.ac.kr.*

**Supplementary Meterials and methods**

**Preperation for EBIBB program**

Harvested air-dried RS was milled using a MF 10 milling machine (IKA, Staufen, Germany). Milled RS in the 425–710-μm range was dried in a vacuum-drying oven at 45°C, and then the resulting solid content was found to be over 97.0% (w/w). In order to reduce various parameters (especially growth rate and pH), similar to confirmed procedures via statistical methodologies (especially Placket-Burman design and Box-Behnken design) (ref. 1), target optimization was carried out in EBIBB program. As a result, the top 3 target component for optimized process were determined as yeast extract, KH_2_PO_4_ and CaCl_2_. Under the EBIBB condition, the extracellular value of MnP predicted approximately 2,800 U/L. Its activity from the experiments was confirmed in good agreement with the predicted values.

**Extracellular activity of well-known lignocellulolytic enzymes**

Regarding a unit (IU, international units) of all enzymes, the fundamental index was defined as the amount of enzyme that releases 1 μmol of target (monomer equivalent) per min.

Similar to previously confirmed manuals (ref. 1, ref. 2), the activity of extracellular MnP was determined by detecting the formation of a Mn (III)-malonate complex at 270 nm after incubating mixtures containing 0.1 mM H_2_O_2_ and 0.2 mM MnSO_4_ at 30°C. In case of lignin peroxidase activity, the oxidative activation in fungal system was determined by the detection of veratraldehyde at 310 nm (ref. 3, ref. 4). In addition, aryl-alcohol oxidase was measured by incubating the mixture containing 2.5 mM veratryl-alcohol in 20 mM sodium succinate (pH 3–4) at 30 ^o^C, and then the oxidation profiles were checked at 310 nm (ref. 3, ref. 5, ref. 6). Furthermore, the presence of glyoxal oxidase was analyzed by monitoring the formation of H_2_O_2_ based on a modified peroxidase-coupled assay (ref. 3, ref. 7, ref. 8).

Based on the broadly confirmed procedure (ref. 9), β-glucosidase was determined by monitoring the release of p-nitrophenyl from p-nitrophenyl-β-D-glucoside at 400 nm for 5 min. Furthermore, the extracellular power of cellobiose dehydrogenase (CDH) was measured by the reduction of *cytochrome c* (12.5 μM) at 550 nm in the presence of cellobiose (100 μM). Here, *cytochrome c* activity of CDH was obtained through 2,6-dichlorophenolindophenol activity (1: 1.54 unit). Lastly, in order to assay xylanase activity, 1 mL mixture containing 0.5 mL of diluted supernatant of broth and 0.5 mL of 1–2% birchwood xylan (Sigma-Aldrich, St. Louis, MO, USA) in 0.05 M sodium citrate buffer (pH 4.8) was analyzed at 50 °C for 30 min, and then the amount of hydrolyzed reducing sugars were checked by the DNS method (http://www.nrel.gov/biomass/analytical_procedures.html) and HPLC.

**Determination of industrial downstream indexes**

Both digestibility (Eq. 1) and fermentability (Eq. 2) were indicated as a percentage of the theoretical maximum of substrate obtained from raw material. Hydrolysis was performed by subjecting samples to 60 FPU of cellulase (Celluclast 1.5L, Sigma-Aldrich, St. Louis, MO) and 30 CBU of β-glucosidase (Novozyme 188, Sigma-Aldrich) per gram of glucan at pH 4.8 at 50 °C and 150 rpm for 120 h. Next, simultaneous saccharification and fermentation using substrates with 3.1% (w/v) glucan in 250 mL of medium was performed using *Saccharomyces cerevisiae* D5A (ATCC 200062) as well as 30 CBU of β-glucosidase and 15 FPU of cellulase per gram of glucan at an initial pH of 5.0. Samples were cultured at 38 °C and 150 rpm for 144 h.

$\text{Glucose yield (\% theoretical maximum) }=\text{ }\frac{\text{g of glucose by HPLC}}{\text{g of glucan}\times1.1}\times100$ (Eq. 1)

$\text{Ethanol yield (\% theoretical maximum) } = \text{ }\frac{\text{g of ethanol}}{\text{g of glucan }\times1.1\times0.511}\times100$ (Eq. 2)

**RNA preparation**

After six biological replicates, fungal communities were collected by filtering through a 0.2-μm PVDF filter (Whatman, Brentford, UK) and transferred in liquid nitrogen. RNA was isolated from the pellets by using 0.2 g zirconia/silica beads (0.5–1 mm, Biospec Products, Bartlesville, OK) and a Mini Beadbeater (Biospec) with 1 mL TRIzol reagent (Invitrogen, Carlsbad, CA). Amplified RNA was obtained using a MessageAmp II-Biotin Enhanced Single Round Amplification Kit (Ambion, Austin, TX) (ref. 10). In order to support weak points (especially low quality and contamination) of the purification, an RNeasy kit (Qiagen, Valencia, CA) was used at the same time. RNA purity was checked by measuring the ratio of absorbance at 230, 260, and 280 nm in a ND**-**1000 spectrophotometer (Thermo Fisher Scientific, Wilmington, DE).

**Probe matching for array design**

In order to confirm a correlation of genetic profiles between RP78 (ATCC 20696, by U.S. Department of Energy Joint Genome Institute (DOE JGI); http://genome.jgi.doe.gov/genome-projects/pages/projects.jsf?searchText=phanero) and EBIBB organism (ATCC 32629), the expression test with RP78-probes (from RP78 genome map) were carried out and then confirmed a sign of “no problem” in significant expression, except for some genes (i.e., below 1%). We ignored the uncertain errors and unknown targets (by DOE JGI), and then significant targets were selected for array-chip.

**Computational statistics-based microarray and data analysis**

Hybridization was carried out at 45^o^C for 16 h with gentle rotation. After washing, biotin labeling was conducted with gentle rotation for 30 min. The arrays were then washed with 2× PBS, 0.1% Tween-20 for 1 min, and 2× PBS for 1 min. The hybridized microarrays were scanned at PMT voltages of 500–700, a pixel size of 5, and a focus position of 130 using a GenePix 4200A microarray scanner (Axon Instruments, Union City, CA). After the data extraction, the background was calculated for individual samples using factory-built control probes with low intensities (5–30%), and their median signal intensities were determined for background subtraction. Microarray datum for each sample was normalized by global normalization using probes with signal values greater than zero. A total of 5,621 probes (except for either unknown function or overlap; actually over 10,000 genes) with signal values lower than maximum 60,000 and those values higher than 5% of the lowest signal value of each sample were subjected to final analysis. Student’s *t*-test was applied to determine differentially expressed sets of genes across 2 experimental groups—EBIBB and NC. Statistical significance was adjusted using the Benjamini-Hochberg FDR multiple testing correction (ref. 11). Avadis Prophetic ver. 3.3 (http://avadis.strandgenomics.com/) was used as the statistical software.

**Quantitative real-time PCR analysis**

Aliquots of cDNA were added to microtiter plates, which was performed with a sequence detection system ABI-PRISM 7900HT (Applied Biosystems, Foster City, CA). The cDNA synthesis was performed using 500 ng RNA at 42°C, and the resultant cDNA was diluted 1:2 prior to use for quantitative real-time PCR. For PCR, 90 nM of primer(s), 250 nM of fluorescence-labeled TaqMan probe, and 5 μl of Universal Master Mix (Applied Biosystems) were used. Finally, 2 μl of cDNA template was added to the reaction mixture. Amplification was performed at 95°C for 10 min for template denaturation, followed by 40 cycles at 95°C for 0.25 min and 60°C for 1 min. The PCR efficiency of targets was reasonably high with 2^n^ copies/cycle. The relative quantities of mRNA were calculated by the 2^–∆∆Ct^ equation (ref. 12).

**Supplementary References**

1. Bak, J. S., Ko, J. K., Choi, I. -G., Park, Y. -C. et al., Fungal pretreatment of lignocellulose by *Phanerochaete chrysosporium* to produce ethanol from rice straw. *Biotechnol. Bioeng.* 2009, *104*, 471–482.
2. Perie, F., Gold, M., Manganese regulation of manganese peroxidase expression and lignin degradation by the white rot fungus *Dichomitus squalens*. *Appl. Environ. Microbiol.* 1991, *57*, 2240–2245.
3. Teunissen, P. J. M., Field, J. A., 2-Chloro-1,4-dimethoxybenzene as a novel catalytic cofactor for oxidation of anisyl alcohol by lignin peroxidase. *Appl. Environ. Microbiol.* 1998, *64*, 830–835.
4. Tien, M., Kirk, T. K., Lignin-degrading enzyme from *Phanerochaete chrysosporium*: purification, characterization, and catalytic properties of a unique H_2_O_2_-requiring oxygenase. *Proc. Natl. Acad. Sci. USA* 1984, *81*, 2280–2284.
5. Bourbonnais, R., Paice, M. G., Veratryl alcohol oxidases from the lignin-degrading basidiomycete *Pleurotus sajor-caju*. *Biochem. J.* 1988, *255*, 445–450.
6. Muheim, A., Waldner, R., Leisola, M. S. A., Fiechter, A., An extracellular aryl-alcohol oxidase from the white-rot fungus *Bjerkendera adusta*. *Enzyme Microb. Technol.* 1990, *12*, 204–209.
7. Kersten, P. J., Kirk, T. K., Involvement of a new enzyme, glyoxal oxidase, in extracellular H_2_O_2_ production by *Phanerochaete chrysosporium*. *J. Bacteriol.* 1987, *169*, 2195–2201.
8. Orth, A. B., Denny, M., Tien, M., Overproduction of lignin-degrading enzymes by an isolate of *Phanerochaete chrysosporium*. *Appl. Environ. Microbiol.* 1991, *57*, 2591–2596.
9. Bao, W., Lymar, E., Renganathan, V., Optimization of cellobiose dehydrogenase and β-glucosidase production by cellulose-degrading cultures of *Phanerochaete chrysosporium*. *Appl. Microbiol. Biotechnol.* 1994, *42*, 642–646.
10. Van Gelder, R. N., von Zastrow, M. E., Yool, A., Dement, W. C. et al., Amplified RNA synthesized from limited quantities of heterogeneous cDNA. *Proc. Natl. Acad. Sci. USA* 1990, *87*, 1663–1667.
11. Benjamini, Y., Hochberg, Y., Controlling the false discovery rate: a practical and powerful approach to multiple testing. *J. R. Statist. Soc.* 1995, *B57*, 289–300.
12. Livak, K. J., Schmittgen, T. D., Analysis of relative gene expression data using real-time quantitative PCR and the 2(-Delta Delta C(T)) Method. *Methods* 2001, *25*, 402–408.

**Supplementary** **table legends**

**Table S1.** Information for quantitative real-time PCR of selected targets.

**Table S1. Information for quantitative real-time PCR of selected targets.**

| JGI ID  (Interpro ID) | Molecular function^a^ | Primer (F/R) | Amplicon  (bp) | Fold^b^ | | | |
| --- | --- | --- | --- | --- | --- | --- | --- |
|  |  |  |  | Arrary | | Real-time PCR | |
|  |  |  |  | NC^c^ | EBIBB | NC^c^ | EBIBB |
| fgenesh1_pg.C_scaffold_5000279  (IPR000254) | Cellulose-binding domain | GTGTCTTCGACTAGCACAAGCA  /TCCTCGACTGCACGAATGAAG | 67 | 3.0 ± 0.3 | 3.3± 0.4 | 7.5 ± 1.3 | 6.5 ± 1.2 |
| e_gwh2.6.371.1  (IPR002016) | Haem peroxidase | CTCGGCGCGGTGACT  /GGTCTCCAGGGCACGT | 83 | 3.9 ± 0.3 | 3.8 ± 0.5 | 2.8 ± 0.9 | 2.5 ± 1.0 |
| e_gwh2.2.798.1  (IPR005829) | Sugar transporter | GTGAGAAGCTCAAAGACTTTGCA  /GAAGCTGAGGCCCATGGA | 80 | 15.0 ± 1.3 | 16.3 ± 2.0 | 2.4 ± 0.8 | 4.4 ± 1.6 |
| e_gwh2.2.56.1  (IPR000608) | Ubiquitin-conjugating enzyme | GCCACGAAGCACTTGCA  /CGCCTTGCGGGTGATGA | 83 | -2.1 ± 0.2 | -2.3 ± 0.3 | -2.2 ± 0.5 | -2.9 ± 0.8 |
| e_gwh2.3.386.1  (IPR007216) | Cell differentiation/sexual development, Rcd1-like | ACACGTCCGTTTGAATATCTTCGT  /CCGTACTGTTGTCATTTTGCTTGA | 79 | -3.4 ± 0.3 | -3.1 ± 0.4 | -12.5 ± 3.0 | -10.4 ± 2.4 |

^a^ the function of the targets was assigned by the DOE JGI.

^b^ ratio of RS-cultures to the control without the substrate.

^c^ negative control; biodegradation without the irradiation.
